# Mthfr deficiency and SARS‐CoV‐2 infection synergistically deplete dopamine and upregulate α‐synuclein increasing the risk of Parkinson's disease

**DOI:** 10.1002/alz70855_103716

**Published:** 2025-12-23

**Authors:** Meenakshi Umar, Saifudeen Ismael, Gregory J Bix

**Affiliations:** ^1^ Department of Neurosurgery, Clinical Neuroscience Research Center, Tulane University School of Medicine, New Orleans, LA, USA; ^2^ Tulane Brain Institute, Tulane University, New Orleans, LA, USA; ^3^ Department of Neurology, Tulane University School of Medicine, New Orleans, LA, USA

## Abstract

**Background:**

Methylene tetrahydrofolate reductase (MTHFR) is a critical enzyme in one‐carbon metabolism that affects several biological processes such as nucleotide synthesis, amino acid homeostasis, DNA methylation, redox defense, and neurotransmitter synthesis including dopamine. Common MTHFR polymorphisms increase the risk of vascular dementia and neurodegenerative diseases such as Parkinson's disease (PD) and are also associated with high COVID‐19 incidence and mortality. Furthermore, Mthfr‐deficient mice display cognitive deficits, cerebrovascular dysfunction, and a increased risk of PD‐like pathology. We hypothesized that SARS‐CoV‐2 infection could accelerate the onset or progression of vascular dementia and PD in the context of MTHFR deficiency as modeled by Mthfr heterozygous knockout (*Mthfr* ^+/‐^) mice.

**Methods:**

We infected 10‐months‐old *Mthfr^+/‐^* and wild type (WT) mice with mouse‐adapted (MA10) strain of SARS‐CoV‐2 (1 × 10^4^ PFU) or with mock (PBS). Mice were monitored for weight loss and euthanized 3 days post‐infection (dpi). Brain and lung tissue were evaluated for viral titer, inflammation, blood‐brain‐barrier (BBB) permeability, and PD pathology. Untargeted metabolomics analysis was performed on plasma samples.

**Results:**

*Mthfr^+/‐^* and WT mice showed comparable weight loss and lung viral titer but no detectable virus in the brain following MA10 infection. Interestingly, we observed increased BBB disruption marked by reduced tight junction protein ZO‐1 and increased BBB leakage in *Mthfr^+/‐^* MA10‐infected mice. MA10 infection also increased IL‐1β mRNA and microglia count in brains of *Mthfr^+/‐^* mice. Metabolomics analysis showed significantly lower plasma dopamine *in Mthfr^+/‐^* infected mice. In addition, MA10 infection significantly decreased tyrosine hydroxylase (TH) level and reduced TH+ dopaminergic neuron count, and increased α‐synuclein levels in substantia nigra of *Mthfr^+/‐^* mice and reduced TH levels in the dorsal striatum.

**Conclusions:**

SARS‐CoV‐2 infection leads to BBB dysfunction**,** neuroinflammation, and lower plasma dopamine levels in *Mthfr^+/‐^* mice. It also decreases dopaminergic neurons in substantia nigra and dorsal striatum, while enhancing α‐synuclein levels in the substantia nigra. These effects may increase the risk of PD, particularly in the context of Mthfr deficiency.

